# Association of prior alcohol misuse with cognitive impairment and Alzheimer’s disease

**DOI:** 10.1002/alz.092066

**Published:** 2025-01-09

**Authors:** Mohammad Housini, Zhengyang Zhou, Sid E. O'Bryant, Nicole Phillips, Robert Barber, Renato A Polimanti, Gita Pathak

**Affiliations:** ^1^ University of North Texas Health Science Center, Fort Worth, TX USA; ^2^ Yale University, New Haven, CT USA; ^3^ Yale School of Medicine, West Haven, CT USA

## Abstract

**Background:**

Alcohol use is hypothesized to be a risk factor for cognitive impairment (CI) and Alzheimer’s disease (AD). However, its role is often complex in diagnosing and distinguishing alcoholic dementia from AD. Hence, several AD‐focused studies exclude participants with possible alcohol misuse history, creating major challenges to investigate this connection. Given that alcohol use is a prevalent yet modifiable factor, it is crucial to study their connection to provide key insights.

**Method:**

We investigated the association of alcohol misuse history with CI and AD in three major AD studies: Health and Aging Brain Study‐Health Disparities (HABS‐HD, N = 3,035), Texas Alzheimer’s Research Care Consortia (TARCC, N = 14,655), and Alzheimer’s Disease Neuroimaging Initiative (ADNI, N = 2,430). Each study collected different metrics of prior alcohol misuse. In HABS‐HD, we used total Alcohol Use Disorder Identification Test (AUDIT) score to define alcohol misuse, while prior alcohol misuse was collected as part of medical history in TARCC and ADNI cohorts. CI was defined as presence of any type of mild cognitive impairment, and AD was defined by each of the respective cohort. We performed the association using regression and two covariate models: Model 1: age, sex, and self‐reported race‐ethnicity; Model 2: Model1+education. To improve statistical power, we performed a meta‐analysis of the three cohorts using fixed‐effects and random‐effects model.

**Result:**

We meta‐analyzed the results from the three for a total sample size of 19,331; 5,345 individuals with AD, 4,820 individuals with CI, and 603 individuals with prior alcohol misuse. We found an association of prior alcohol use with AD (Model 1: OR = 1.87; 95CI = 1.55‐2.26; p‐value = 7.99×10^−11^; Model 2: OR = 1.75; 95CI = 1.33‐2.29; p‐value = 5.0×10^−5^), and CI (Model 1: OR = 1.47; 95CI = 1.21‐1.77; p‐value = 6.23×10^−05^; Model 2: OR = 1.39; 95CI = 1.06‐1.84; p‐value = 1.75×10^−02^). There was no heterogeneity among the cohort‐specific effects contributing to the meta‐analysis. (heterogeneity p‐value> 0.05).

**Conclusion:**

Leveraging multiple AD cohorts, we show that prior alcohol misuse is associated with CI and AD, warranting further inquiry into possible molecular and biological processes that intersect between AD and alcohol abuse/dependence.

